# Assessment of community’s knowledge, attitude and practice about onchocerciasis and community directed treatment with Ivermectin in Quara District, north western Ethiopia

**DOI:** 10.1186/1756-3305-7-98

**Published:** 2014-03-10

**Authors:** Fitsum Weldegebreal, Girmay Medhin, Zemichael Weldegebriel, Mengistu Legesse

**Affiliations:** 1 Department of Medical laboratory Science, Haramaya University, College of Health and Medical Science, Harar, Ethiopia; 2 Aklilu Lemma Institute of Pathobiology, Addis Ababa University, Addis Ababa, Ethiopia; 3 Metema Hospital, Gendwuha, North Gondar, Amhara Region, Ethiopia

## Abstract

**Background:**

The African Program for Onchocerciasis Control (APOC) has been working with ultimate goal of reducing the public health and socio-economic problems associated with onchocerciasis within a period of 12–15 years. Although dedicated community engagement is crucial for the success of the program, there is little/no information on the levels of community’s knowledge, attitude and practice about onchocerciasis as well as about the ongoing control program in Ethiopia. In this study, we have assessed the level of knowledge, attitude and practice of Quara district residents about onchocerciasis and the current control strategies in the area.

**Methods:**

This community-based cross-sectional study was conducted between October 2012 and January 2013 in Quara District, Amhara Regional State, North West of Ethiopia. The study participants were recruited from randomly selected kebeles (small administrative units) of the study area and were interviewed about onchocerciasis and about community directed treatment with ivermectin (CDTI**)** using structured questionnaire. The collected data were double entered into a data entry file using EpiData software, V.3.1. The data were transferred to SPSS soft-ware V.16 and analyzed according to the different variables.

**Results:**

Out of 418 respondents, 401 (95.9%) of the respondents have heard about onchocerciasis (locally known as ‘wara’) and 11.2% said that they knew about the etiology of the disease, which was named as filarial worm. However, 356 (88.8%) had at least one misconception about the causative agent of onchocerciasis. More than half (69.4%) knew that the transmission of the disease is related to black fly biting. Overall, 93.3% participants believed that onchocerciasis is preventable, of whom 49.5% indicated use of drug as the means of preventing the disease. Majority (95.5%) of the participants perceived CDTI as very useful program.

**Conclusion:**

Although onchocerciasis is endemic disease in the study area, large proportion of the community had conspicuous misconceptions in all issues about its causation, transmission and preventive methods. This could affect the success of the CDTIP in the present study area. Therefore we recommend increasing the awareness about onchocerciasis in the area through community-based campaigns during drug distribution with especial focuses on females and age group less than 35 years”.

## Background

Onchocerciasis is a disease caused by *Onchocerca volvulus (O.volvulus).* The disease is characterized by causing skin lesions with severe itching, a serious eye lesion and blindness known as river blindness. The disease is a major problem among rural communities living in close proximity to rivers in Sub-Saharan African countries, Latin and Central America [1]. It spreads by the biting of black flies belonging to the genus Simulium which breed in fast flowing rivers.

The ultimate goal of the African Program for Onchocerciasis Control (APOC) is to reduce the public health and socio-economic problems of onchocerciasis within a period of 12–15 years using the strategy of yearly community-directed treatment with ivermectin (CDTI) in endemic areas. Population-based chemotherapy program using ivermectin is highly effective, feasible and offers a different approach to the control of onchocerciasis [2,3]. Ivermectin, when taken annually, has the ability to bring about sustained reduction in skin and eye microfilariae to very low levels with reduction in morbidity [4,5] and transmission [6,7].

A complete national survey (1997–2004) in Ethiopia indicated that onchocerciasis was Endemic in nine regions, with 7.3 million people at risk and more than 3 million already infected. The prevalence of onchocerciasis in Ethiopia ranges from 6.9% in the Quara District of Northwest Ethiopia to 85.3% in Teppi, South western Ethiopia [8].

Community is the heart of the APOC strategy for controlling onchocerciasis. Community ownership of the distribution program has been a major innovation for mass treatment, and has been a corner stone to the success of CDTI. However, the full participation of the community could be largely affected by several factors including low level of community’s awareness about the disease, community’s attitudes towards the program, system of drug distribution and the motivation of community drug distributors (CDDs) [9]. A community-based free distribution of ivermectin was first launched in Sheka Zone, Southwestern Ethiopia in the year 2001 and then, in the rest of affected regions it was launched phase by phase [10]. CDTI program was introduced to Quara District in 2003 by WHO/APOC in partnership with Federal Ministry of Health (FMOH), the Carter Centre, the local administration and the communities. All eligible members of the community in the District have been treated with ivermectin in campaign once a year. Pregnant women, less than one week of lactating mothers, seriously ill individuals and under five children are not eligible. Except individuals mentioned above, any individual living in the selected area is eligible for the CDTIP [11].

Ignorance and wrong beliefs about the disease can lead to negligence in prevention and control measures and it causes accepting inappropriate treatment. Involvement of individuals and communities is an important component of onchocerciasis control activities. To attain community participation and design socially/locally acceptable control strategies, health program planners and implementers must be familiar with people’s knowledge and attitude in relation to onchocerciasis [12]. The successful use of ivermectin at national, regional, zonal, District and kebeles requires a broad public health program designed to ensure appropriate distribution, monitoring, community education, and record keeping. There is paucity of information as few studies have been carried out to understand these issues. Therefore, a study aimed at assessing factors affecting the sustainability of the program has great importance to generate information on the awareness, challenges and obstacles faced during ivermectin distribution, and design appropriate strategy to improve the outcomes. The finding of this study will serve as an input for the Quara District Health Office while designing monitoring and evaluation of the on-going onchocericiasis control program. The information obtained from the study provides a basis for understanding how best to sustain community control and to achieve success in the control of onchocerciasis as a public health and socioeconomic problem in the study area. However, the knowledge of the communities about onchocerciasis and their attitude towards the CDTIP has not been studied in the present study area. Therefore, the aim of this study was to investigate people’s knowledge and beliefs about onchocerciasis and their attitudes towards the CDTIP in Quara area, North Western Ethiopia.

## Methods and materials

### Description of the study area and population

Between October 2012 and January 2013, a community- based cross sectional survey was conducted in Quara District, North western Ethiopia, which is about 1041 kilometers North of Addis Ababa and 324 kilometers North-west of Gondar town. The District has a total area of 858,580 square kilometers and it shares geographical boarder with Metema District in the North, Benshangul Regional State in the South, North Sudan in the West, Alefa District in East and Awi zone in South West. There are three major ethnic groups in the District (i.e. Aguw, Amhara and Gumuz) and there are also minorities who come from Tigray, Oromiya and the Southern Nations Nationalities and Peoples Region (SNNPR). Based on the 2007 national housing and population census [13], the District has 19 kebeles (small administrative unit) with a total population of 93,629, consisting of 49,750 men and 43,879 women. The District has 20, 806 households with an estimated density of 4.50 person per square kilometer [13]. CDTI program was introduced to Quara District in 2003 by WHO/APOC in partnership with FMOH, the Carter Centre, the local administration and the communities with 100% geographic coverage [11].

Out of the 19 kebeles of the District 17 kebeles were occupied by the Amhara ethnic group and the other 2 kebeles were occupied by the Aguw and Gumuz ethnic groups. In the District, there were 5 health centers and 28 health posts, giving routine services for the population. Onchocerciasis is one of the major public health problems in the District [11] with the prevalence of 6.9% [8].

### Sample size estimation and data collection

There was no previous information on the level of community’s knowledge, attitude and practice about onchocerciasis in the present study area. Hence, it was hypothesized that at least 50% of adult residents of the target area (i.e. 18 years of age or above) would have a good level of knowledge about the disease (i.e. they will score above the mean value within a given sample). Hence, sample size was estimated taking this as the starting point with 95% confidence and 5% degree of accuracy. Since the source population is very mobile, sample size was increased by 10% to compensate for non-respondents resulting in the final sample size of 422. The participants were eligible if they were a member of the selected kebele, age 18 and above years, apparently healthy and willing to volunteer to participate in the study.

Two out of the 17 kebeles occupied by Amhara ethnic group were randomly selected, while each kebele of the Aguw and Gumuz ethnic groups were purposely included. Based on the number of people aged 18 years and above in each kebele, the pre-estimated sample size of 422 was proportionally distributed. Ivermectin treatment registration book of each kebele was used as the sampling frame. Study participants were recruited using systematic random sampling. In case the selected individual was absent in the first and second visit**,** the next individual on the list whose age was 18 years or above was included. Structured questionnaires were prepared in English based on information from available literatures (9; 12; 14; 15) and the questionnaires were translated into Amharic and pre-tested for clarity and cultural acceptability in the district. The participants were interviewed in their local languages by trained data collectors (health extension workers) who speak the local languages. Each interview was made by house-to-house visit. Information on the socio-demo-graphic characteristics of the participants was also included in the questionnaires.

### Ethical consideration

The study protocol was approved by the Ethical Clearance Committee of Aklilu Lemma Institute of Pathobiology (ALIPB), Addis Ababa University. Permission was obtained from Quara District Health Office and from the four selected kebeles administrators. Participants were informed about the objective of the study and they were assured the confidentiality of the data to be maintained. Informed verbal consent was obtained from all participants prior to data collection.

### Data analysis

The collected data were double entered into a data entry file using EpiData software, V.3.1. The data were transferred to SPSS soft-ware V.16 and analyzed according to the different variables. Pearson chi-square was used to evaluate the statistical significant of bivariate association of selected covariate. Odds ratio with 95% CI generated using logistic regression were used to describe the strength of association between the selected study variables (i.e. outcome and independent variables) before and after controlling for possible confoundering variables. Bivariate and multivariable logistic regression analysis was performed to explore independent variables that were predictors of overall knowledge (causative agents, sign/symptoms, mode of transmission, treatment and preventive methods of onchocerciasis), attitude and practice of the people on onchocerciasis and CDTIP. The correct answer was coded as 1 and wrong answer was coded as 0. To generate the overall knowledge, attitude and practice score of all correct responses were added. Respondents whose knowledge, attitude and practice scores equal and above the mean were considered as having ‘good knowledge, attitude and practice’ while those below the mean were considered as having ‘poor knowledge, attitude and practice’. The criterion for significance was set at P < 0.05 based on a two-sided test.

## Results

### Socio-demographic characteristics of the study participants

From the random community sample of 422, we were able to attain response rate of 99.1% and female respondents constituted of 37.3%. The age of the respondents ranged from 18–87 years, with mean age of 35.2 and SD of 11.7 years. The majority were in the age range of 25 to 49 years, and was belonged to Amhara ethnic group, farmers and followers of Orthodox Christianity (Table 1).

**Table 1:** Socio-demographic characteristics of 418 study participants recruited from the community, Quara District, 2013

**Characteristics**	***Number *** **(%)**	**Characteristics**	***Number *** **(%)**
Kebele		**Religion**	
Yikaho	63(15.1)	Orthodox Christian	337(80.6)
Mahdid	62(14.8)	Muslims	81(19.4)
Bambaho	126(30.1)	**Educational level**	
Dubaba	167(40)	Illiterate	248(59.3)
**Gender**		Primary (grades 1–8)	142(34.0)
Male	262(62.7)	Secondary and above	28(6.7)
Female	156(37.3)	**Occupation**	
**Age (years)**		Farmer	349(83.5)
18-24	67(16.0)	Others	69(16.5)
25-49	303(72.5)	**Family size**	
50-64	35(8.4)	1-4	230(55.0)
65+	13(3.1)	5+	188(45.0)
**Ethnicity**			
Amhara	293(70.1)		
Agew	63(15.1)		
Gumuz	62(14.8)		
**Marital status**			
Unmarried	48(11.5)		
Married	365(85.2)		
Others	14(3.3)		

### Knowledge, attitude and practice of the community about onchocerciasis

The most important identifying perceptions of the community which hinder the uptake of preventive and treatment services in the district were: they were at field (farming) during the campaign day; CDDs were not coming to their house to provide them with the treatment, they believed that freely given medications are useless for health and they feared side effects of the drug. The level of knowledge, attitude and practice of respondents is summarized in Tables 2, 3 and 4. Out of 418 respondents, 95.9% have heard about onchocerciasis (locally known as ‘wara’) and 11.2% said that they knew about the etiology of the disease, which was named as filarial worm. On the other hand, 88.8% had at least one misconception about the causative agent of onchocerciasis including black fly biting (58.1%), poor personal hygiene, and living in poor environmental sanitation, eating contaminated food, and witchcraft. More than half of the respondents (58.6%) mentioned that onchocerciasis can be transmitted and most of them (69.4%) knew that the transmission of the disease is related to black fly biting. The remaining suggested contact with a person who has the disease, mosquito bite, sharing cloths and through breathing as mode of transmission. Majority (78.1%) of the participants mentioned itching as a symptom of the disease, while 42.6% and 26.7% mentioned skin change and edema as a symptom of onchocerciasis, respectively. Three hundred seventy seven (94.0%) knew that onchocerciasis is treatable disease.

**Table 2:** Knowledge of community respondents (n = 418) about onchocerciasis, Quara District, 2013

**Indicative questions on knowledge**	**Response categories**		**Number (%)**
Have you ever heard about the disease called onchocerciasis	Yes		401(95.9)
	No		17(4.1)
Causative agent of onchocerciasis	Filarial worm		45(11.2)
	Black fly		233(58.1)
	Mosquito		14(3.5)
	Living in poor environmental sanitation		59(14.7)
	Poor personal hygiene		33(8.2)
	Witchcraft		2(0.5)
	Eating contaminated food		1(0.2)
	Being not vaccinated		9(2.2)
	Do not know		5(1.2)
Oncho transmits from person to person	Yes		235(58.6)
	No		120(29.9)
	I do not known		46(11.5)
The mode of transmissions of the disease	Black fly bite		163(69.4)
	Contact with infected person		40(17.0)
	Mosquito bite		19(8.1)
	Through breath		1(0.4)
	Sharing clothes		9(3.8)
	I do not know		3(1.3)
The signs and symptoms of the disease	Itching	Yes	313(78.1)
	Edema	Yes	107(26.7)
	Skin change	Yes	171(42.6)
Do you think oncho is preventable disease	Yes		374(93.3)
	No		11(2.7)
	I do not know		16(4.0)

**Table 3:** Attitude and practice of community respondents (n = 418) towards onchocerciasis, Quara District, 2013

**Indicative questions on attitude and practice**	**Response categories**	**Number (%)**
Have you/your families ever been sick from onchocerciasis	Yes	126(31.4)
	No	267(66.6)
	I do not remember	8(2.0)
Is onchocerciasis a serious disease	Yes	386(96.3)
	No	10(2.5)
	I don’t know	5(1.2)
Do you think onchocerciasis need treatment	Yes	377(94.0)
	No	12(3.0)
	I do not know	12(3.0)
Type of treatment used	Modern	370(98.1)
	Traditional	7(1.9)
If Modern, which drug is needed to treat the disease	Ivermectin/Mectizan	327(88.4)
	Albendazole	43(11.6)
What do you do to prevent onchocerciasis	Avoiding river bathing	204(54.5)
	Wearing protective clothes	153(40.9)
	Taking drug	185(49.5)
	Using bed net	141(37.7)
	Environmental sanitation	93(24.9)
	Personal hygiene	53(14.2)
If your answer for the above question is Wearing protective clothes, in what way is used	In the lower extremities (below the knees)	124(81.0)
	Around head & shoulders	29(19.0)

**Table 4:** Attitude and practice of community respondents about CDTI, Quara District, 2013

**Indicative questions on attitude and practice**	**Response**	**Number (%)**
How do you/your family perceive CDTI	Very useful	399(95.5)
	Partially useful	16(3.8)
	Not useful	2(0.5)
	I do not know	1(0.2)
What is your contribution in the CDTI	Taking the drug continuously	409(97.8)
	I do not know	9(2.2)
Do you think the program on controlling Onchocerciasis is effective	Yes	399(95.5)
	No	7(1.7)
	I do not know	12(2.9)
What do you recommend to continue the program	Drug supply	195(46.7)
	Transport	42(10.0)
	Incentive for CDDs	180(43.1)
	No comment	1(0.2)
Have all eligible family members received ivermectin	Yes	333(79.7)
	No	85(20.3)
If your answer is no, who missed the treatment	wife	44(51.8)
	Husband	34(40.0)
	Children	7(8.2)
How many times did he/she or you miss the treatment	One year	71(83.5)
	Two years	14(16.5)
Why did he /she or you missed treatment	Pregnancy	37(43.5)
	Health problem	41(48.3)
	Not present during drug distribution	7(8.2)
Do you know who interrupted the treat treatment in the village	Yes	126(30.1)
	I do not know	292(69.9)
If your answer is yes, What was the reason to interrupt the treatment	Side effect of drug	68(50.7)
	Lack of good case management in side effect	66(49.3)
When did you received your last	last year	397(95.0)
Treatment	Before two years	21(5.0)
Did the drug have any side effects	Yes	163(39.0)
	No	248(59.3)
	I don’t know	7(1.7)

Among the total respondents, 386 (96.3%) said that onchocerciasis is a serious disease and 374 (93.3%) believed that onchocerciasis is preventable disease. Among the total respondents, 370 (98.1%) knew that the disease is treated with modern medicine and from these respondents 327 (88.4%) knew the name of the drug used to treat the disease i.e. ivermectin/mectizan. Regarding preventive methods, 50.8% suggested avoiding river bathing, 49.5% mentioned taking drug, and 40.9% mentioned wearing protective cloths and 37.7% mentioned use of bed net. About 31.4% of the respondents reported that they/their families had got the disease (Table 3).

**Figure 1:**
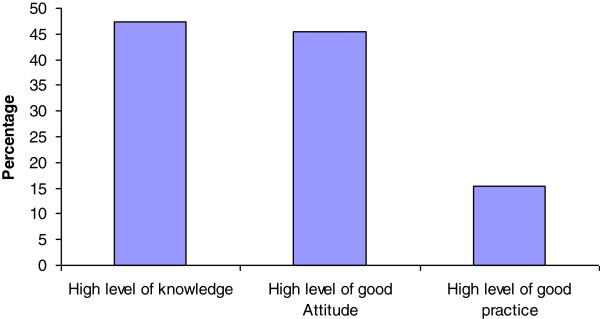
**Level of community’s knowledge, good attitude and good practice of towards onchocerciasis, Quara District, 2013.** Each percentage represents fraction of total respondents who have scored above mean score within each category of the three outcomes.

From the total respondents, 190 (47.4%), 182 (45.4%) and 62 (15.5%) had good level of knowledge, attitude and practice towards onchocerciasis, respectively (Figure 1).

### Community’s knowledge, attitude and practice about CDTI

All of the respondents knew that CDTIP has been initiated in their kebeles. Among the respondents, 350 (83.7%) knew that CDDs distribute the drug and 398 (95.2%) knew that CDDs distribute the drug during week end or holidays. Among the respondents, 266 (63.6%) mentioned that the drug was distributed properly and 315 (75.4%) knew that the drug has other useful effect, of whom 285(90.5%) mentioned that the drug used to treat intestinal parasitic infections. Majority of the respondents (83.7%) mentioned that the drug was distributed house to house. Among the respondents, 87 (20.8%) knew that the treatment period is decided by the community, but others knew that the treatment period is decided by the District health Office (48.6%), CDDs (16%), HEWs (12.2%) and community leaders (2.2%).

Among the respondents, 409 (97.8%) knew their responsibility on the program and 395 (95.5%) perceived CDTI as very useful program, but 16 (3.8%) perceived the program as partially useful. For sustainability of this program, 195 (46.7%) recommended continuous drug supply, while 180 (43.1%) recommended incentive for CDDs. The majority (79.7%) responded that all eligible family members took the drug annually, whereas 85(20.3%) responded that family members missed at least one round of the drug due to health problems, pregnancy and absence during drug distribution. To facilitate the late memory to the reaction of the drug, the respondents were asked ^#^when did they receive the last treatment^$^, 397(95%) of the respondents responded last year (Table 4).

**Figure 2:**
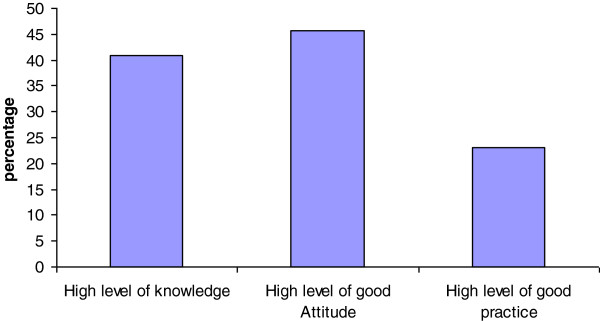
**Level of knowledge, good attitude and good practice of community about CDTI, Quara District, 2013.** Each percentage represented fraction of total respondents who have scored above the mean score within each category of the three outcomes.

From the total of 422 respondents 171(40.9%) had good level of knowledge, 191(45.7%) had positive attitude and 96 (23.0%) and had good practice towards CDTIP (Figure 2).

### Results from Bivariate analysis

In bivariate analysis, ethnicity was the only factor associated with community’s knowledge, attitude and practice towards onchocerciasis: Participants who belong to Amhara ethnic group were about 4 times more likely to have good level of knowledge compared to participants from Agewu ethnic group (crude OR = 4.44, P < 0.001, 95% CI = 2.11, 9.35), and about 9 times more likely to have good level of knowledge compared to participants from Gumuz ethnic group (crude OR = 9.17, P < 0.001, 95% CI = 4.18, 20.00). With regard attitude towards the disease, participants from Amhara ethnic group were 4 times more likely to have good level of positive attitude compared to participants from Agewu ethnic group (crude OR = 4.44, P < 0.001, 95% CI = 2.14, 9.26), and 27 times more likely to have good level of positive attitude compared to participants from the Gumuz ethnic group (crude OR = 27.03, P < 0.001, 95% CI = 9.71, 76.92). Similarly, being Amhara ethnic group was 3 times more likely to have good level of positive practice compared to that of Agewu ethnic group (crude OR = 2.68, P = 0.031, 95% CI = 1.12, 8.40), and 7 times more likely to have good level of positive practice compared to Gumuz ethnic group (crude OR = 6.76, P = 0.005, 95% CI = 1.78, 25.64) (Table 5).

**Table 5:** Results of bivariate analysis about onchocerciasis

**Variable**		**Crude odds ratio, 95% CI**	**p-value**
**Ethnicity**			
**Knowledge**	Agew	1	**<0. 001**
	Amhara	**4.444** (2.114, 9.346)	
	Gumuz	1	**<0. 001**
	Amhara	**9.174** (4.184, 20.00)	
**Attitude**	Agew	1	**<0.001**
	Amhara	**4.444** (2.141, 9.259)	
	Gumuz	1	**<0.001**
	Amhara	**27.027 **(9.709, 76.923)	
**Practice**	Agew	1	**0.031**
	Amhara	**2.681** (1.115, 8.403)	
	Gumuz	1	**0.005**
	Amhara	**6.757** (1.783, 25.641)	

Regarding practice towards CDTIP, male respondents were about 2 times more likely to have good practice compared to female respondents (crude OR = 1.77, P = 0.033, 95% CI = 1.05, 2.99). On the other hand, none of the examined factors were significantly associated with high level of knowledge or high level of positive attitude towards the disease or the program (Table 6).

**Table 6:** Rresult of Bivariate Analysis of Community Respondents about CDTI

**Variables**		**Crude odds ratio, 95% CI**	**p-value**
**Practice**	Sex		
	Female	1	0. 033
	Male	**1.767**(1.046, 2.985)	

## Discussion

Majorities of the study participants were familiar with onchocerciasis; this is probably due to the endemicity of the disease in the study area. In the area, the disease is called as ‘wara’ which means ‘itching skin disease’. However, many authorities believe that low knowledge and practice of the peasants of rural Africa predispose them to infection [14]. In this study, from 418 subjects, only 45 (11.2%) knew about the etiology (causative agent) of the disease and the majority held at least one misconception about the cause of onchocerciasis which is consistent with the findings of other studies (9; 12; 17). On the other hand, the majority of the respondents associated the causative agent of the disease with the bite of black flies, which is almost comparable to the finding of study conducted in Bebeka, Southwest Ethiopia [12]. Similarly, in this study, majority of the participants held at least one misconception about mode of transmission of onchocerciasis which is consistent with the findings of the study conducted in Bebeka, Southwest Ethiopia [12].

Majority of the study subjects believed that onchocerciasis is a serious disease. This finding is also in agreement with the findings of the studies conducted in Bebeka Southwest Ethiopia [12] and Sequa area, Southwest Ethiopia [15]. However, majority of the study participants had good knowledge about sign and symptoms of the disease; this is probably due to the endemicity of the disease in the study area and the finding is consistent with the findings of the study conducted in Sequa area, Southwest Ethiopia [15].

Generally, in this study, majority of the study participants had poor level knowledge of onchocerciasis (i.e. only 45.5% of the participants had good level of knowledge). This finding is also consistent with the findings of the study conducted in Sequa area, Southwest Ethiopia [15]. Similarly, majority of the study subjects had poor attitude and practice about onchocerciasis (i.e. only 45.4% and 14.8% study subjects had good attitude and good practice on onchocerciasis, respectively) which is probably due to shortage of health education at the community level and the CDDs may not be properly trained about onchocerciasis due to negligence of health extension workers to supervise the CDDs in delivering health education, and/or excluding the community interventions for onchocerciasis in the health extension package.

Community ownership of the distribution program has been a major innovation for mass treatment, and has been a corner stone to the success of CDTI. The full participation of the community could be largely affected by the drug reaction compared to endemicity of the disease [16]. In this study, the full participation of the community could be largely affected by the drug reaction, CDDs were not coming to their house to provide them with the treatment, they believed that freely given medications are useless for health and they feared side effects of the drug. This observation is consistent with the studies conducted in Okpuje, an endemic community in Edo State, Nigeria [17]. In this study, almost all of the participants knew ivermectin is very important for significant reduction in morbidity and take the drug annually properly. This observation is consistent with the studies conducted in a hyper-endemic community of Edo State, Nigeria [9], and Ethiopia [15] and in Imo State, Nigeria [18]. However, a few individuals interrupted the treatment due to fear of drug related adverse reactions which is similar to the findings of studies conducted in an endemic area of Guatemala [19], in Okpuje, an endemic community in Edo State, Nigeria [17], in the hyper-endemic community of Edo State, Nigeria [9] and in Sequa area, Southwest Ethiopia [15].

In addition to this, they knew that the drug has other useful effect including treating intestinal parasitic infections. The finding is consistent with the findings of the study conducted in North East Nigeria [20] and Sequa area, Southwest Ethiopia [15].

In this study, majority of the study participants mentioned that the coverage of ivermectin distribution was 100%, which is necessary for significant and persistent regression in morbidity. This coverage is better in magnitude compared to the findings of the study conducted in Sequa area, Southwest Ethiopia study [15]; this is probably due to the long period (ten years) distribution of the drug in the present study area. The limitations of this study were: the study was not supported by qualitative methods like focus group discussions and epidemiological studies. In spite of this limitation, this study provides an important information regarding knowledge, attitude and practice of community towards onchocerciasis and CDTI chemotherapy for onchocerciasis control, identifying perceptions of the community which hinder the uptake of preventive and treatment services in the district, and base line information for the Quara District Health Office for planning, monitoring and evaluation of the on-going onchocericiasis control.

## Conclusion

In conclusion, though many people in the study communities are familiar with onchocerciasis, most of them lack information on the correct causative agent, mode of transmission and prevention of onchocerciasis with conspicuous misconceptions in all issues. This could affect the success of the CDTIP in the present study area. Hence, this study revealed the need for increasing the awareness about onchocerciasis in the area through community-based campaigns during drug distribution with especial focus on females and age group less than 35 years is very important for the effectiveness of the program. This will important to improve acceptance and support of the CDTI. Development of health education materials should focus on causative agent, mode of transmission, and prevention of onchocerciasis information in order to ensure better understanding of individuals about the disease.

## Competing interests

The authors declare that they have no competing interests.

## Authors' contributions

FW designed the study, participated in data collection, analysis, interpretation, and write-up, drafted the manuscript and critically revised the manuscript. ML participated in study design, critically revised the manuscript. GM participated in study design, and critically revised the manuscript. ZW participated in data collection and analysis and interpretation. All authors read and approved the final manuscript.
